# Nirvana: A Qualitative Study of Posttraumatic Growth in Adolescents and Young Adults with Inflammatory Bowel Disease

**DOI:** 10.3390/children9060879

**Published:** 2022-06-13

**Authors:** Qiwei Wu, Pingting Zhu, Xinyi Liu, Qiaoying Ji, Meiyan Qian

**Affiliations:** 1 School of Nursing, Yangzhou University, Yangzhou 225009, China; mz120201760@yzu.edu.cn (Q.W.); mx120190879@yzu.edu.cn (X.L.); mz120201761@yzu.edu.cn (Q.J.); mx120211164@stu.yzu.edu.cn (M.Q.); 2 Jiangsu Key Laboratory of Zoonosis, Yangzhou 225009, China

**Keywords:** adolescents and young adults, inflammatory bowel disease, posttraumatic growth, a qualitative study

## Abstract

(1) Background: Psychosomatic discomfort is prevalent among adolescents and young adults (AYAs) with inflammatory bowel disease (IBD). Post-traumatic growth (PTG) has been a protective factor in earlier research. However, little is known regarding PTG among AYAs with IBD. This study investigates the generation of PTG in adolescents and young adults with inflammatory bowel disease (IBD) and finds positive coping skills employed in clinical nursing practice. (2) Methods: In 2021, 32 individuals were interviewed utilizing a semi-structured interview guide. This study used qualitative content analysis. (3) Results: The interviews revealed five themes: spiritual change, internalized supportiveness, cognitive re-shaping, externalized behaviors, and future-oriented thinking. (4) Conclusions: The research revealed the presence of PTG in AYAs with IBD. To give tailored care to patients, medical professionals must monitor the state of their PTG development in a planned and focused manner.

## 1. Introduction

Inflammatory bowel disease (IBD) is a type of chronic immune-mediated disease whose etiology is unknown and includes ulcerative colitis (UC) and Crohn’s disease (CD) [1]. It is characterized by recurring bouts for which there is no permanent cure. Intestinal symptoms (e.g., diarrhea, abdominal pain) and extraintestinal symptoms (e.g., joint pain, fatigue) have severely compromised the health and quality of life of IBD patients [2,3]. However, existing treatment methods, including medication and surgery, can only alleviate but not cure the illness [4,5,6]. Over the past 20 years, the incidence rate has risen rapidly in China due to changes in economic status, lifestyle, and diet [7].

The disease primarily affects adolescents and young adults (AYAs), who are at a critical stage of development in practically every aspect of their lives, including their social, academic, and personal development [8]. As a result of the disease attacking AYAs repeatedly, they are forced to seek treatment everywhere. Thus, AYAs are often forced to make tough choices that interfere with their everyday lives, such as taking sick leaves, missing school, or dropping out of school to face long-term treatment [9]. Being plagued by symptoms and having a severe reduction in quality of life leads to psychological comorbidities such as anxiety, depression, and post-traumatic stress disorder [10]. Although the patient has been suffering from the condition for a long time, good coping feelings, known as (PTG), arise, according to previous research [11]. PTG is a positive psychological change associated with an individual in a challenging life environment or adverse event (e.g., illness, bereavement) [12]. The study of PTG has, for the most part, been centered on adults and focused on natural disasters, critical illness, and terrorism [13]. In recent years, research on PTG among AYAs has sprung up. According to Fraus’s research, most teenagers who experienced many traumas attributed their PTG experiences to a single incident [14]. IBD patients are more likely to attribute post-traumatic stress (PTSS) to their disease [15]. Klosky studied the relations between PTSS and PTG in long-term survivors of childhood cancer and proposed that PTSS may be a prerequisite for PTG [16]. Tedeschi came up with a framework for post-traumatic growth, posited that post-traumatic stress and growth are related, and proposed that PTSS may be a prerequisite for PTG [17]. Although subsequent studies proved a positive correlation between PTSS and PTG, a cross-sectional study design could not prove a causal relationship [18,19]. Existing studies have shown that AYAs with cancer have higher PTG levels. An investigation of AYAs who had survived cancer revealed moderate to high levels of PTG [20]. Fractal dimension analysis of PTG reveals that AYAs show significant changes in “appreciation of life” and “relationships with others” but show a low level of “spiritual change” [20,21]. In conclusion, AYAs diagnosed with IBD are likely the driving force behind the formation of PTGs. Due to the unique characteristics of AYAs during the growth stage, the production of PTG will serve as a potential protective factor. Therefore, it is crucial to investigate the PTG formation mechanism in AYAs. 

Most existing studies use quantitative methods to describe patients’ PTG levels [22]. By emphasizing meaning and understanding, qualitative research complements quantitative research, answering questions opaque to quantitative research [23]. Qualitative research focuses on the real feelings of the research object in a specific situation. Its purpose is to understand the process of events, situations, experiences, and actions and their significance to the research object [24]. It is considered to be the best way to experience things or processes. 

In addition, researchers discovered that healthcare providers (HCPs) acted primarily as educators and healers while interacting with IBD patients, such as by advising them on how to control symptoms [25], providing counseling to improve the quality of life for these patients, and monitoring their disease status with follow-ups [26]. There is a shortage of focus on the psychological components of the IBD population, particularly with regard to positive psychology. Statistically, between 30 to 50 percent of IBD patients require further psychological assistance, although outpatient settings do not routinely provide psychological support [2]. Only one of the seventeen research studies included in a recent systematic review of factors impacting resilience in young individuals with IBD addressed resilience as an objective, and none of the studies contained validated measures of resilience [27]. 

No detailed research on the PTG levels of AYAs diagnosed with IBD has been conducted. Thus, the results of this qualitative study are given in a narrative format to better investigate how AYAs experience PTG after being diagnosed. Notably, the findings of this study may equip HCPs with evidence-based knowledge regarding how to improve care for AYAs with IBD.

## 2. Materials and Methods

### 2.1. Design

This research adopted the descriptive phenomenology method, which is a method that reflects the nature of the thing [28]. We generated open-ended question guidelines for the interview based on a literature study and team discussion. This study was conducted in line with the Helsinki Declaration.

### 2.2. Participants and Setting

Thirty-two AYAs diagnosed with IBD participated in this study. Since this study focused on a specific demographic, recruitment was conducted using the technique of purposive sampling. After evaluating the patients’ medical records, the first author personally asked the eligible patients if they were willing to participate in this study. For patients younger than 18 years old, parental consent is required. The literature demonstrates that teenagers older than 15 are generally as capable as adults in making medical decisions [29]. Therefore, an interview with AYAs is feasible. The inclusion criteria for patients were as follows: (i) patients who have been diagnosed with IBD, (ii) 15–25 years old, (iii) clear consciousness and normal hearing and speaking, (iiii) understand the purpose of this study and volunteer to participate. We stopped the recruitment when all the authors agreed that the interview information saturated (no new content appeared). The participants’ demographic characteristics are presented in Table 1.

### 2.3. Ethical Considerations

The Yangzhou University School of Nursing Ethics Committee accepted the study (Identification code: YZUHL2021008, Approved by 15 March 2021). The objectives of this study were communicated to all participants via an informed consent form and face-to-face conversation. Before inviting minors to participate in the study, the legal guardian’s consent was sought. Only the first and corresponding authors have access to the passwords for the encrypted electronic folder containing participants’ personal information and interview transcripts.

### 2.4. Data Collection

The study data were collected from April to August 2021. The data collection used a semi-structured interview from two hospitals in Yangzhou, Jiangsu Province. A semi-structured interview is a standard mode in phenomenological research that is useful in understanding the experience and psychological process of the participants [30]. Each interview was conducted for approximately 60–90 min by two researchers who have a doctor’s degree and a master’s degree, respectively, in the meeting room of the ward. 

### 2.5. Data Analysis

This study employed a qualitative content analysis method [31]. The interview data were transcribed within 24 h. To ensure accurate transcription, the participants were asked to recite the content. We extensively analyzed the data using the appropriate approach. Two researchers independently read, extracted, and coded each recorded transcript. The codes were derived directly from the transcription, which is the basis for the initial coding scheme. Then, the researchers categorized the codes into themes and subthemes based on the relationships and connections between the codes. The entire study team engaged in regular and ongoing conversations to ensure that the conceptual meanings and vocabulary were appropriate. Consensus was used to present all findings. 

## 3. Results

**Figure 1:**
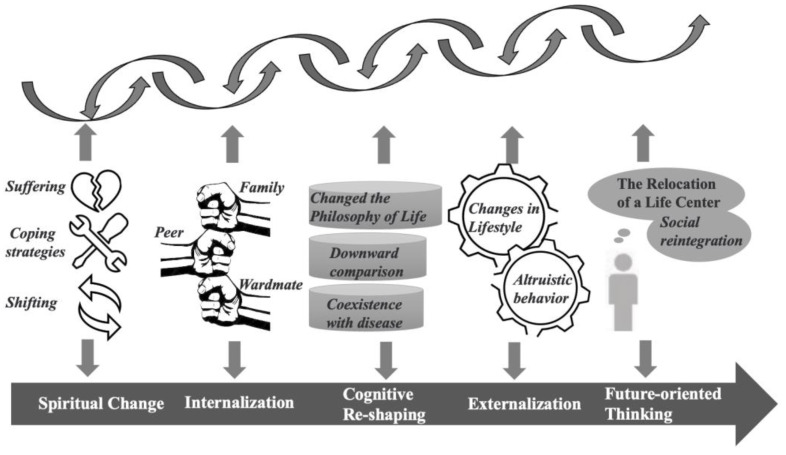
The conceptual model of the generation of post-traumatic growth.

Five themes were captured during the process of the production of PTG. Figure 1 (see Figure S1) depicts the study results. The generation of PTG is gradual (straight arrow) or tortuous (curvilinear arrow).

### 3.1. Theme 1—Spiritual Change

When first diagnosed with IBD, almost all patients (n = 28, 90%) felt that a black hole had swallowed up their lives. As they struggled with the disease, their feelings changed. We divided this change into three sub-themes: suffering negative emotional experiences, seeking emotional coping strategies, and shifting into positive emotions.

#### 3.1.1. Suffering from Negative Emotions

It is common to experience negative emotions after a diagnosis. In the early stages of illness, all participants interviewed reported feeling negative emotions.

Participant 18: “When I knew there was no cure for IBD, my world collapsed. I locked myself in the room every day and didn’t want to face it”. 

One participant mentioned that their parents had emotionally impacted him or her.

Participant 27: “I did not know well about IBD, but my parents’ reaction to the disease scared me”.

#### 3.1.2. Seeking Emotional Coping Strategies

Participants consciously adjusted their emotions whenever they realized they were about to be dominated by negative emotions. Coping strategies were employed during the adjustment process.

Participant 2: “I know my mood is volatile; I can only do the best to adjust; watching TV is a better effective strategy to stop thinking”.

Participant 19: “Once I get into a state of self-entanglement, I will force myself not to think or find something else to do to divert my attention”.

#### 3.1.3. Shifting in Positive Emotions

More than half of the participants (*n* = 19, 61%) indicated that their negative emotions were relieved as they were converted to positive emotional experiences after adopting the emotional strategies. Furthermore, some patients (*n* = 13, 42%) found a correlation between the positive emotional experience and the slowing down of the disease’s development. Findings like these reinforced their desire to seek positive psychological experiences.

Participant 8: “I want to make myself feel better. Because I found that mood is magical, when I am in a good mood, my bowel symptoms will improve accordingly. My doctor also told me that they were related”.

Only a few patients (n = 5, 16%) reported that HCPs explained the relationship between emotions and symptoms. Professional explanations and advice enable them to maintain a positive emotional state to prevent symptoms.

Participant 24: “The doctor keeps telling me to keep in a good mood…maintaining a positive mood is what the doctor ordered”.

### 3.2. Theme 2—Internalize Supportiveness

After diagnosis and throughout treatment, participants reported receiving multimodal social support. From the perception of social support to the continual internalization of these forces to enhance the inner self, it fosters and solidifies the growth of PTG. We grouped this support into three sub-themes based on the participants’ description: family support, peer support, and wardmate support.

#### 3.2.1. Family Support

Approximately two-thirds of the participants (*n* = 18, 58%) said that seeking family support was the first response to asking for help when they learned of the illness. They thought that the comfort and encouragement of the family were the most accessible and the most basic forms of support.

Participant 30: “Without a doubt, I will have the support of my family at first. I do not need to express anything to them, and they will take the initiative to assist me”.

In addition to psychological support, most participants mentioned that the family’s dietary structure would change to cater to their eating habits.

Participant 16: “There will be no dishes that I can’t eat at the dinner table”. 

Participant 23: “My mother prepares lunch boxes for me to take to school every day”. 

#### 3.2.2. Peer Support

One-quarter of the participants (*n* = 8, 26%) said they would talk to their friends and colleagues about their concerns but could not elaborate on the details of their illness due to its complexity. In the realm of peer support, it is more about seeking companionship.

Participant 21: “IBD is too complicated. I don’t know how to describe it in detail because I don’t think they can empathize with me. Chatting and singing karaoke with me would be good for me”. 

Some participants (*n* = 11, 35%) expressed fear of being ostracized but felt relieved that their peers understood.

Participant 5: “I’m grateful that my classmates didn’t look at me differently”.

#### 3.2.3. Wardmate Support

Most participants (*n* = 27, 87%) expressed their willingness and hoped to join the wardmate club. “Holding together for warmth” was the most frequently used term. This group is particularly lonely as IBD is not shared. Consequently, they hope to get more experiences from others with the same disease to help them progress step by step.

Participant 13: “When I was diagnosed, I was pulled into a chat group, a group of wardmate. I felt like I’d found my place. They described their experiences and made me feel strong enough to live”.

### 3.3. Theme 3—Cognitive Re-Shaping

When producing PTG, many negative concepts (e.g., unfortunately, the end of life) in the mind are re-forged. Cognition is re-shaped in multiple ways. Three sub-themes are included: coexistence with the disease, downward comparison, and change in the philosophy of life.

#### 3.3.1. Coexistence with Disease

When participants (n = 29, 90%) realized that IBD was a lifelong condition, attitudes toward the disease began to change. Due to the fact that the illness is unchangeable, they can only change their mentality to accept this fact.

Participant 16: “It took me long to accept that the disease was lifelong. But all my changes are based on the fact that I have to accept the truth”.

#### 3.3.2. Downward Comparison

Repeated emotional distress is a standard mentality change described by participants after the illness. A few participants (*n* = 6, 19%) reported that downward comparison relieved their negative emotions. A downward comparison was used in the process of comparison to obtain positive emotions.

Participant 7: “I admit that I have been in a negative mood at times. Compared to those in a worse situation than mine, or when I heard about some unfortunate events, I found that I was doing quite well”.

#### 3.3.3. Changed the Philosophy of Life

The experience of illness allowed the participants (*n* = 17, 53%) to re-examine their lives, from perceiving life to reworking life cognition.

Participant 11: “My life has changed since I got sick. Every person has only one life, and I want to live it better”.

Participant 24: “It made me realize that I had to live for myself…My life needed variety”.

### 3.4. Theme 4—Externalized Behaviors

Participants reported behavioral as well as psychological changes. Changes in behavior result from inner emotions; therefore, externalizing causes them. The changes can be categorized into two sub-themes: changes in lifestyle and altruistic behavior.

#### 3.4.1. Changes in Lifestyle

IBD-afflicted AYAs must passively adapt their lifestyle behaviors, such as nutrition and sleep. Initially, some participants (n = 10, 31 percent) claimed that practicing these behaviors (eating habits were most frequently mentioned) made them angry and upset. Nonetheless, when individuals developed a better understanding of IBD and received encouragement from outside sources, their inner feelings and behaviors changed for the better.

Participant 10: “Since I have controlled my diet and routine, I have a regular life schedule much better than before”.

Participant 14: “I used to enjoy drinking and barbecuing with my friends, but now I don’t eat junk food consciously”.

#### 3.4.2. Altruistic Behavior

More than half of the participants said that their own experience and experiences in fighting the disease more or less resonated with their fellow wardmates and hoped they would be able to help others.

Participant 9: “It is not easy for someone who has IBD. I am willing to provide emotional counseling and disease guidance to people who have just been diagnosed”.

### 3.5. Theme 5—Future-Oriented Thinking

A definitive diagnosis can be a source of anxiety for participants, but progressive acceptance of the illness and external support can provide them with a sense of future direction. We extracted two sub-themes: the relocation of a life center and social reintegration. 

#### 3.5.1. The Relocation of a Life Center

IBD participants reflected on the past and planned for the future due to their suffering. According to some, the illness changed the center of their lives.

Participant 20: “I used to put my career first, giving up my health for work. It’s not too late to realize that health is the most important thing”.

#### 3.5.2. Social Reintegration

Studying and working is an essential part of the future for all AYA participants because they do not want to be separated from society by IBD.

Participant 1: “Because I have been taking sick leave all the time, so I have to quit my job. I still want to work in the future…I don’t want to be out of touch with society”.

## 4. Discussion

This study’s objective was to investigate the process of PTG production among AYAs diagnosed with IBD and to offer a foundation for how HCPs can support PTG. 

IBD is still considered a rare disease in China [32], so patients diagnosed with IBD cannot accept that such a small probability event happened to them. Retrospective memories are activated at the moment of learning of a diagnosis, as described by the participants. Negative mood swings result from persistent doubts about the cause of the disease and regrets over past habits [33,34]. While our research was underway, we also discovered that signs of PTG could also be observed shortly after IBD was diagnosed.

We discovered that the increase in PTG output is gradual and tortuous. AYAs frequently reflect on the disease before sensing assistance and gaining direction in the event narrative. Two sorts of meditating can be distinguished: intrusive and deliberate ruminating [35]. Previous studies have reported a negative correlation between invasive rumination and PTG, but deliberate rumination may promote PTG [36,37]. HCPs must identify and guide patients to distinguish between the different types of rumination. After being diagnosed, HCPs are one of the most frequently used sources of support for AYAs [38]. A recent HCP-administered mindfulness intervention improved depression in AYAs with IBD [39]. These findings support the value of HCP-led interventions to promote PTG in AYAs. In addition, the brain-gut axis plays a connecting role in symptoms and negative emotions in patients with IBD [40,41]. Interfering with patients is a two-way street, and the starting point cannot be symptoms or unpleasant emotions alone. However, few participants stated that their health care providers described this reciprocal relationship to them, which may result from HCPs’ insufficient awareness of how emotions affect lives, as reported by AYAs. HCPs must act swiftly to guide AYAs’ positive thinking and expedite their entry into the PTG development zone. 

The external support AYAs receive is multidimensional, including internal family structures and social networks. A few AYAs disclose to peers and gain support [42]. Rather than receiving peer support, AYAs are more concerned about avoiding exclusion. Because they may be more susceptible to ridicule and have a higher need for social acceptance than adults with IBD, this is consistent with previous research indicating that AYAs with this disorder are prone to feeling lonely and disconnected from peers. We also discovered that not everyone views their family as their primary support system. Some AYAs state that they do not wish to alarm their families. They conceal certain realities, such as aspects of their sickness and psychology, and pick other forms of social support. In this study, most participants selected a wardmate as one of their external supports because, in the specific state of shared IBD, the individual qualities of these individuals in clusters vanish, and they exist as a “group consciousness” [43]. Duncan presented a model that showed that personality characteristics and life experiences are related to participation in collective action, both directly and indirectly, through the development of group consciousness [44], which means that every emotion and behavior is contagious. Moreover, when the “group consciousness” is infected with positive psychology as the group’s central theme, the PTG of the group will also coincide [45]. That is to say, those who are at odds with group psychology (or a theme) will be regarded as group heterogeneous and be excluded (e.g., negative psychology). Thus, when a patient with poor mental health status enters a positive group, the group consciousness of that person may be activated to produce PTG. Therefore, group intervention using “group psychology” can promote the generation of PTG [45]. A meta-analysis of 12 studies that measured PTG as the outcome of an intervention concluded that group interventions fostered higher levels of PTG [46]. Even if family support is easier to obtain, AYAs are more likely to select wardmate assistance, and the PTG level demonstrates stability. This can be described as a combined and attracting force. In the future, HCPs may be required to organize patient meetings consistently. This form of intervention, which is not confined to face-to-face or Internet interactions, may promote social bonds, adaptive coping, and the growth of AYAs’ PTG. 

In this study’s theme of “cognitive re-shaping,” cognitive changes in AYAs were shown, including changes in attitudes towards life and disease, and “downward comparison” strategies were mentioned. Cognitive appraisal is an emotion regulation strategy [47,48]. A positive change in consciousness can be achieved by changing or comparing how emotional events are interpreted, thus reducing negative emotions or promoting positive emotions [49]. During cognitive restructuring, AYAs change the experience of unease and even life-related confusion into a sense of control. To complete the transition from “child care” to “adult care,” the provision of care for AYAs is challenging due to the increasing knowledge that can improve physical and mental health outcomes. At this time, HCPs can provide tailored health education ranging from dispelling AYAs’ uncertainty about the disease to spiritual guidance [50,51]. Downward comparison can be an effective strategy to optimize patients with negative emotions, which is reflected in our study’s findings and reported in previous studies [52]. This is because downward comparison enhances appreciation of one’s circumstances and helps sustain motivation and well-being [53]. Tara’s study showed that downward comparison was associated with greater subjective well-being at low levels of perceived control [52]. Thus, this would be a protective strategy for AYAs who are in psychological distress and have a low sense of control of IBD.

The primary objective of coexisting with IBD is to maintain remission for as long as feasible. Behavioral changes also accompany cognitive changes. Dietary and lifestyle modifications are the most prevalent, although some patients exhibit superego adjustments, often known as altruistic conduct. Some academics feel that altruism stems from pain [54]. Others believe it stems from increased empathy. Learning that their feelings were reciprocated during the AYAs’ process of self-disclosure increased their PTG levels. As a result, they are willing to help others, and they find solace in the fact that this is a situation that kills two birds with one stone.

IBD is a lifelong disorder. A positive tendency in the evolution of PTG is the steady expansion from the present to the future of patients’ attention in ongoing self-acceptance. The inability to study and work leads to troubles for AYAs, which is the main content of future planning. To a certain extent, IBD disrupts the original trajectory of their lives and hinders their reintegration into society [54]. Considering the coexistence of diseases, some of the original plans had to be revised. New coping strategies were adopted, such as altering the working environment and using time compensation strategies to make up for the lost time during disease onset and treatment. This is consistent with Calvet’s research that found that disease can affect the type of work taken up or that people lose or reject jobs due to IBD [55]. AYAs are keen to engage in social activities. PTG will benefit from professional career planning assistance. Future research should study therapies at PTG levels associated with AYA IBD, different evolutionary stages, and the role of HCPs as reputable professionals in the prospective rehabilitation process. Then, individualized interventions might be designed to promote a positive reframing of AYAs’ IBD caregiving experiences. 

### Strengths and Limitations

The existing research lacks insight into the PTG level of IBD in AYAs. Open-ended and semi-structured, our interview guide focuses on the topic and emphasizes the subjective experience of AYAs. Qualitative approaches offer this benefit. However, it has limitations as well. First, the age distribution of the participants is imbalanced, with only one-fourth of the participants under the age of eighteen. Thus, there is room for error. Second, participants may have relatively higher PTG levels than those who declined the interview.

## 5. Conclusions

Understanding the mechanism of PTG formation in AYAs with IBD is crucial in China, where the prevalence is rising annually. After the mental and physical trauma they endured after being diagnosed, we detected the shadow of PTG in them, and the progression of PTG was gradual or torturous. According to the findings of this study, HCPs should be guided according to the various stages of PTG development and assisted in overcoming their doubt and disempowerment.

## Figures and Tables

**Table 1:** Demographic characteristics of the participants.

*n* = 32	
Gender	
Male	14
Female	18
Age (years)	
15–17	8
18–25	24
Disease type	
Ulcerative colitis	19
Crohn’s disease	13
Education level	
Less than middle school	2
High school	10
Higher than Undergraduate	20
Time since diagnosis	
Less than one year	7
More than 1–3 years	16
More than three years	9

## Data Availability

The data presented in this study are available on request from the corresponding author.

## Supplementary Materials

The following supporting information can be downloaded at: https://www.mdpi.com/article/10.3390/children9060879/s1, Figure S1: The conceptual model of the generation of posttraumatic growth.
