# Supplementary figures and images for: Nirvana: A Qualitative Study of Posttraumatic Growth in Adolescents and Young Adults with Inflammatory Bowel Disease
Source: Children (Basel). 2022 Jun 13;9(6):879. doi: 10.3390/children9060879 (PMC9222066; doi:10.3390/children9060879)

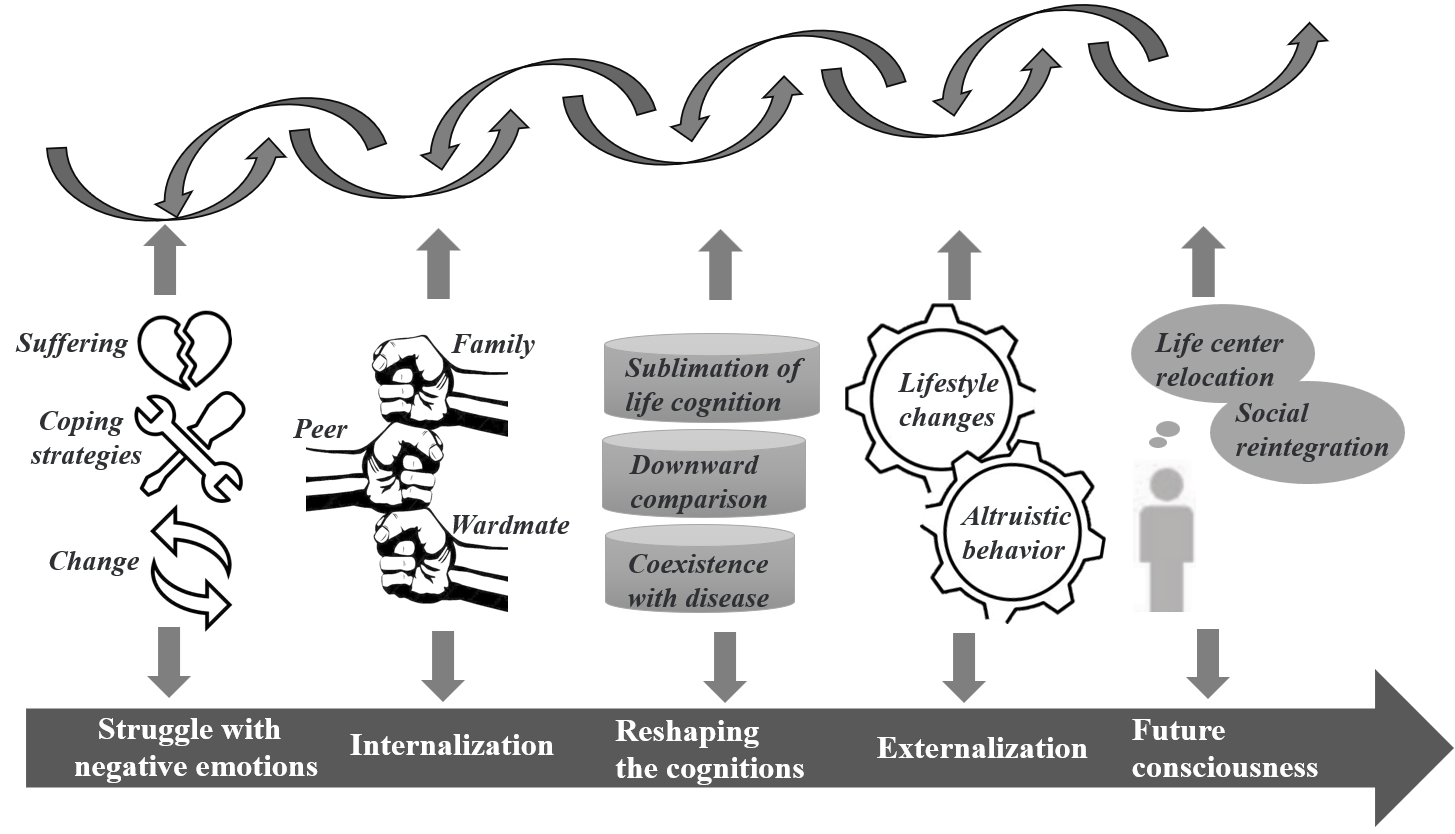

Supplement: Supplementary file 1 [file children-09-00879-s001.zip › children-1696209-supplementary.jpg]
